# Transaxillary Versus Transaortic Transcatheter Aortic Valve Implantation in the Treatment of Aortic Stenosis: An Updated Systematic Review and Meta-Analysis

**DOI:** 10.7759/cureus.24054

**Published:** 2022-04-12

**Authors:** Ishaque Hameed, Mohammad O Khan, Ibtehaj Ul-Haque, Omer M Siddiqui, Syed A Samad, Shanza Malik, Samar Mahmood

**Affiliations:** 1 Internal Medicine, Dow University of Health Sciences, Civil Hospital Karachi, Karachi, PAK; 2 General Surgery, Dow University of Health Sciences, Civil Hospital Karachi, Karachi, PAK

**Keywords:** transcatheter aortic valve implantation (tavi), transaxillary, transaortic, meta-analysis, a systematic review

## Abstract

Transcatheter aortic valve replacement (TAVR) is a technique that can be performed through multiple approaches, and the benefits of one approach over another are still being evaluated to make sure patients receive the best possible care. Our meta-analysis aims to compare clinical and procedural outcomes of the transaxillary (TAx) and transaortic (TAo) approaches to validate the more optimal procedure.

The systematic literature search was done via PubMed/MEDLINE, Embase, and the Cochrane Central databases from inception to December 2021, to identify articles reporting data on both TAx TAVR and TAo TAVR. In addition, we checked ClinicalTrials.gov for more published or unpublished trials. Baseline patient characteristics, procedure results, and clinical results were extracted from the article and pooled for analysis. A quantitative meta-analysis was conducted using Review Manager (RevMan) version 5.3 (Nordic Cochrane Centre, The Cochrane Collaboration, Copenhagen, Denmark). The outcomes extracted included blood transfusion, conversion to sternotomy, tamponade, contrast amount, procedure time, bleeding incidents (minor, major, or life-threatening), length of stay (LOS), vascular complications (minor or major), acute kidney injury (AKI), paravalvular leak (PVL), permanent pacemaker (PPM) implantation, 30-day mortality, one-year mortality, 30-day stroke, and device success.

The final analysis included 11 articles, consisting of 10 observational studies and a pivotal trial. Cumulative results revealed that the TAo approach had a significantly lower incidence of vascular complications (RR = 2.30; 95% CI = 1.22 to 4.35), and the need for implantation of a permanent pacemaker (RR = 1.82; 95% CI = 1.30 to 2.54) along with a lower amount of contrast (mean difference (MD) = 27.40; 95% CI = 3.73 to 51.08) needed to be used. The TAx group was associated with a significantly lower 30-day mortality (RR = 0.46; 95% CI = 0.31 to 0.69), AKI (RR = 0.47; 95% CI = 0.33 to 0.67), and length of hospital stay (MD = −1.95; 95% CI = −2.51 to −1.38). No significant difference was observed between the outcomes of 30-day stroke (RR = 1.38; 95% CI = 0.81 to 2.33), PVL (RR = 1.05; 95% CI = 0.50 to 2.18), tamponade (RR = 0.71; 95% CI = 0.12 to 4.03), conversion to sternotomy (RR = 0.51; 95% CI = 0.06 to 4.30), device success (RR = 0.97; 95% CI = 0.88 to 1.07), the incidence of bleeding (RR = 0.75; 95% CI = 0.51 to 1.10), and procedure time (MD = 4.44; 95% CI = −96.30 to 105.17).

Both the procedures were associated with their benefits and risks. Although most of the outcomes favored TAx transcatheter aortic valve implantation (TAVI), it is too early to say if it would be better than TAo TAVI. To authenticate the findings concluded in this meta-analysis and further improve our understanding of the efficacy, safety, and risk profile between TAx and TAo approaches for TAVI, large sample randomized clinical trials are required on a wide scale.

## Introduction and background

Calcific aortic stenosis (AS) constitutes a significant health problem in the elderly, the prevalence of which is about 8.1% at 85 years of age [1]. After symptoms have developed, the only effective treatment is aortic valve replacement (AVR)/aortic valve implantation (AVI). The American College of Cardiology/American Heart Association (ACC/AHA) guidelines recommend AVR as a Class I indication for severe, symptomatic AS (i.e., the proposed treatment, procedure, or intervention is effective and should be performed for the majority of patients under most circumstances). However, nearly one-third of patients are deemed unsuitable for surgery due to concerns about age, comorbidities, patient frailty, and severe left ventricular dysfunction [2]. Transcatheter aortic valve implantation (TAVI) is a technique that has revolutionized the management of AS and has risen exponentially as the standard of care for patients at prohibitive surgical risk, emerging as a promising treatment for patients of moderate to high-risk levels [3].

As TAVI comes of age, significant variability exists in the alternative access techniques available for its utility. Transfemoral access (TF) dominates as the most preferred route, owing to its less invasive approach, higher rates of survival, and considerably lower complications [4]. However, a considerable proportion of individuals are not eligible for the TF route due to luminal narrowing, atherosclerosis, obstruction, calcification, or tortuosity of the iliofemoral vessels [5]. Despite the progress in the miniaturization of delivery systems, unfavorable anatomy and peripheral vascular disease preclude TF access in approximately 17.2% of patients [6]. Thus, various alternate accesses have been proposed over the years and are in use, of which this article examines in depth two: the transaortic (TAo) and transaxillary (TAx) approaches.

The TAx method uses local anesthetic and mild sedation, followed by a convenient surgical cutdown from the deltopectoral groove to the pectoralis major: dissection or retraction of the pectoralis then yields exposition of the subclavian artery [7,8]. This avoids the invasiveness of other techniques and overcomes peripheral vascular disease [8]. Further, progressive advancement has led to fully percutaneous procedures without surgical cutdown [7], making TAx comparable to the TF approach and potentially, the safer non-TF route. The TAo access is achievable through a mini-sternotomy or a right thoracotomy, allowing exposure of the proximal ascending aorta [8]. This route is advantaged by eluding smaller arteries (iliofemoral or the subclavian) en route by direct insertion of the sheath in the aorta, thus decreasing the risk of complications. Additionally, it employs a highly accurate transfer of the operator’s maneuvers to the delivery system while using safe and easy valve placement [8]. The detailed evaluation of these vascular surgical approaches, in terms of anatomy and technique, has been included by Pascual et al. in their article on the same [8].

Both TAo and TAx approaches offer specific procedural advantages that cannot be ignored. However, the limited documentation in the literature comparing the two accesses does not allow operators to favor one approach over the other. The relative benefits and risks of each are still subject to much debate, indicating the need for them to be better defined so that the choice of one over the other can be fully delineated. Therefore, our study aimed to compare clinical and procedural outcomes of the TAx and TAo approaches such as mortality, 30-day stroke, and acute kidney injury (AKI).

## Review

Methods

Data Sources and Search Strategy

Two independent researchers (I.H. and M.O.K.) searched PubMed, Embase, and Cochrane Central from inception until December 2021. The Preferred Reporting Items for Systematic Reviews and Meta-Analyses (PRISMA) guidelines were used to conduct this meta-analysis [9]. We used the following keywords and terms to search each database: “Transaxillary,” “Trans-axillary,” “Transsubclavian,” “Trans-subclavian,” “Transaortic,” “Trans-aortic,” “Direct aortic,” “Trans-cervical,” “Transthoracic,” “Transcatheter aortic valve implantation,” “Transcatheter aortic valve replacement,” “TAVI,” and “TAVR.” The comprehensive search algorithm for the database is in Appendix A. The reference lists of the retrieved publications and previous meta-analyses were manually screened for potentially relevant studies. In addition, we checked ClinicalTrials.gov for more published or unpublished trials. The term “transaxillary access” was employed instead of “transsubclavian access” because transsubclavian access is regarded as a misnomer unless a supraclavicular cut-down is performed. Furthermore, transsubclavian and transaxillary techniques are interchangeable terms in the literature [10].

Study Selection and Eligibility Criteria

The studies were considered eligible if they were randomized controlled trials (RCTs) or observational studies, irrespective of publication status. In addition, to be included, articles had to report outcomes for mortality, length of stay (LOS), stroke, bleeding, or vascular complications between TAx and TAo transcatheter aortic valve replacement (TAVR). We omitted the studies if the data were insufficient or inadequate for analysis, if the study was a case report or review, or if the study was in a non‑English language.

Data Extraction and Assessment of Study Quality

The articles found through the systematic search were imported into the EndNote Reference Library software (Clarivate, London, UK), identifying and eliminating duplicates. Two separate reviewers (I.H. and M.O.K.) thoroughly reviewed the remaining publications, and only articles that satisfied the previously specified criteria were accepted. Dialogue resolved disagreements, failing which a third reviewer (S.A.S.) was consulted. We extracted the patients' baseline characteristics and diverse set outcomes from the finalized articles, including device success, blood transfusion, conversion to sternotomy, tamponade, contrast amount, procedure time, bleeding incidents (minor, major, or life-threatening), LOS, vascular complications (minor or major), AKI, PVL, permanent pacemaker (PPM) implantation, 30-day mortality, 30-day stroke, and one-year mortality. Furthermore, the Newcastle-Ottawa Scale was utilized to evaluate the quality of observational studies (Appendix B).

Statistical Analysis

Review Manager (RevMan) version 5.3 (Nordic Cochrane Centre, The Cochrane Collaboration, Copenhagen, Denmark) was used to perform all the statistical analyses. We calculated the dichotomous data using RRs with 95% CIs as meaningful effect measures for 30-day mortality, one-year mortality, 30-day stroke, PPM implantation, AKI, PVL, and vascular complications. Some papers reported medians and interquartile ranges transformed to mean and standard deviation using the methods presented by Wan et al. [11]. The random-effects model and the Higgins I² statistic were used for the analysis and heterogeneity calculation. We defined an I² of <50%, 50-75%, and >75% representing low, moderate, and high heterogeneity, respectively. The statistical significance level for hypothesis testing was set at 0.05. We used subgroup analysis for the comparisons between observational studies and RCTs. Furthermore, we performed sensitivity analysis when heterogeneity was >50% or when the same institution or author reported two similar studies; in such cases, we included the more recent publication or the one with the greatest information (Appendix C). A funnel plot for the primary outcome of 30-day mortality was generated to evaluate the possibility of publication bias.

Results

Literature Search and Baseline Characteristics

**Figure 1:**
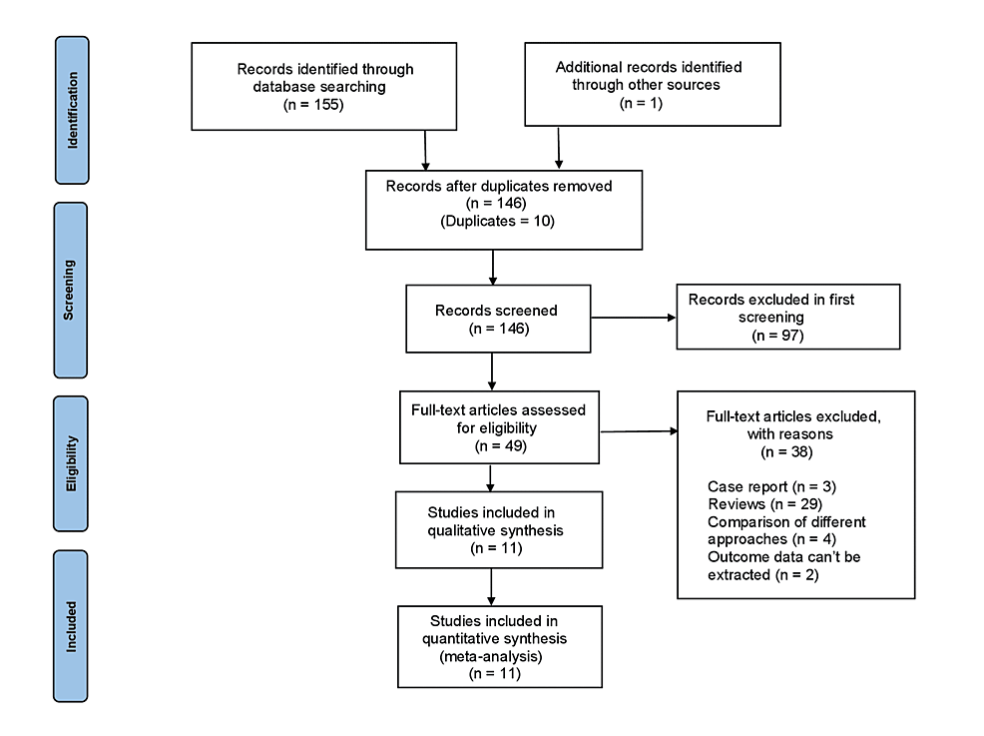
PRISMA flow diagram The PRISMA diagram details the search and selection processes applied during the overview. PRISMA: Preferred Reporting Items for Systematic Reviews and Meta-Analyses.

The initial database search yielded a total of 156 potentially relevant articles. After removing the duplicates, 146 articles were screened for suitability and relevance based on their titles and abstracts. Out of these, 49 full-text articles on the objective of the manuscript were reviewed. After the full-text screening, 36 articles were excluded. Two articles were also excluded during data extraction. The final analysis included 11 articles, consisting of 10 observational studies and a pivotal trial. Figure 1 represents the PRISMA flowchart, outlining our systematic review’s search and screening process. The baseline characteristics are included in Table 1.

**Table 1:** Baseline characteristics of the included studies TAo = transaortic; TAx = transaxillary; N/R = not reported; BMI = body mass index; HTN = hypertension; DM = diabetes mellitus; LVEF = left ventricular ejection fraction; MI = myocardial infarction; COPD = chronic obstructive pulmonary disease; AF = atrial fibrillation; NYHA = New York Heart Association; EuroSCORE = European System for Cardiac Operative Risk Evaluation; STS PROM = Society of Thoracic Surgeons Predicted Risk of Mortality.

Study	Year	Region	Approach (n)	Age, mean (SD)	Gender (M:F)	BMI, mean (SD)	HTN	DM	LVEF, mean (SD)	Prior MI	COPD	Prior AF	Prior cardiac surgery	NYHA III/IV	Peripheral vascular disease	Logistic EuroSCORE		STS PROM		Newcastle-Ottawa Scale
																Mean (SD)	P-value	Mean (SD)	P-value	
Myat et al. [12]	2020	UK	TAx (82)	78.3 (6.8)	54:28	27.6 (5.4)	N/R	24	N/R	11	27	N/R	23	N/R	47	N/R	N/R	N/R	N/R	8/9
			TAo (142)	80 (8.9)	69:73	27.2 (6.4)	N/R	37	N/R	34	43	N/R	26	N/R	77	N/R		N/R		
Lin et al. [13]	2021	N/R	TAx (56)	N/R	N/R	N/R	N/R	N/R	N/R	N/R	N/R	N/R	N/R	N/R	N/R	N/R	N/R	N/R	N/R	7/9
			TAo (11)	N/R	N/R	N/R	N/R	N/R	N/R	N/R	N/R	N/R	N/R	N/R	N/R	N/R		N/R		
Pineda et al. [14]	2019	USA	TAx (30)	N/R	N/R	N/R	N/R	N/R	N/R	N/R	N/R	N/R	N/R	N/R	N/R	N/R	N/R	N/R	N/R	7/9
			TAo (24)	N/R	N/R	N/R	N/R	N/R	N/R	N/R	N/R	N/R	N/R	N/R	N/R	N/R		N/R		
Beve et al. [15]	2019	France	TAx (73)	N/R	N/R	N/R	N/R	N/R	N/R	N/R	N/R	N/R	N/R	N/R	N/R	N/R	N/R	N/R	N/R	7/9
			TAo (41)	N/R	N/R	N/R	N/R	N/R	N/R	N/R	N/R	N/R	N/R	N/R	N/R	N/R		N/R		
Codner et al. [16]	2018	USA	TAx (11)	84 (5.1)	2:9	27.2 (9.1)	11	3	59.5 (12.8)	1	N/R	N/R	2	10	5	N/R	N/R	7.6 (2.1)	0.499	7/9
			TAo (11)	83 (4.3)	7:4	25.4 (5.5)	11	1	54 (14.9)	3	N/R	N/R	3	11	4	N/R		8.5 (3.8)		
Damluji et al. [17]	2018	France and USA	TAx (17)	80.3 (9.7)	10:7	27.3 (5.6)	13	4	59.3 (9.7)	1	N/R	5	N/R	7	N/R	N/R	N/R	N/R	N/R	8/9
			TAo (67)	84.3 (5.3)	30:37	25.6 (5.3)	55	19	58.3 (11.3)	17	N/R	21	N/R	38	N/R	N/R		N/R		
Khan et al. [5]	2018	USA	TAx (24)	84.7 (7.9)	13:11	27 (5.2)	22	9	52.2 (17.3)	N/R	6	11	7	22	6	25 (15)	1	8.66 (6)	0.06	8/9
			TAo (27)	82.6 (7)	10:17	26 (6.4)	27	10	55.8 (15.9)	N/R	8	10	8	24	9	25 (16)		11.3 (4)		
Fiorina et al. [18]	2017	Italy	TAx (147)	83 (5)	72:75	N/R	N/R	N/R	51 (12)	N/R	N/R	N/R	22	119	N/R	15.3 (15.7)	0.0001	7.3 (6)	0.006	7/9
			TAo (95)	82 (6)	44:51	N/R	N/R	N/R	52 (14)	N/R	N/R	N/R	25	78	N/R	27.3 (17.3)		9.6 (6.8)		
Fröhlich et al. [19]	2015	UK	TAx (188)	82.3 (5.9)	123:65	26 (4.4)	N/R	45	N/R	52	52	32	63	N/R	N/R	23.3 (14.9)	0.891	N/R	N/R	8/9
			TAo (185)	83 (8.2)	90:95	25.6 (4.4)	N/R	39	N/R	43	71	30	44	N/R	N/R	23.1 (14.9)		N/R		
Adamo et al. [20]	2015	Italy	TAx (32)	82 (6)	14:18	25 (4)	23	8	49 (13)	5	5	15	4	24	21	26.3 (10.1)	0.906	9.3 (6.5)	0.412	8/9
			TAo (44)	83 (6)	27:17	25 (6)	29	15	48 (15)	7	3	6	10	35	31	26 (11.5)		8.23 (4.83)		
Reardon et al. [21]	2014	N/R	TAx (146)	N/R	N/R	N/R	N/R	N/R	N/R	N/R	N/R	N/R	N/R	N/R	N/R	N/R	N/R	N/R	N/R	7/9
			TAo (340)	N/R	N/R	N/R	N/R	N/R	N/R	N/R	N/R	N/R	N/R	N/R	N/R	N/R		N/R		

Quality Assessment and Publication Bias

**Figure 2:**
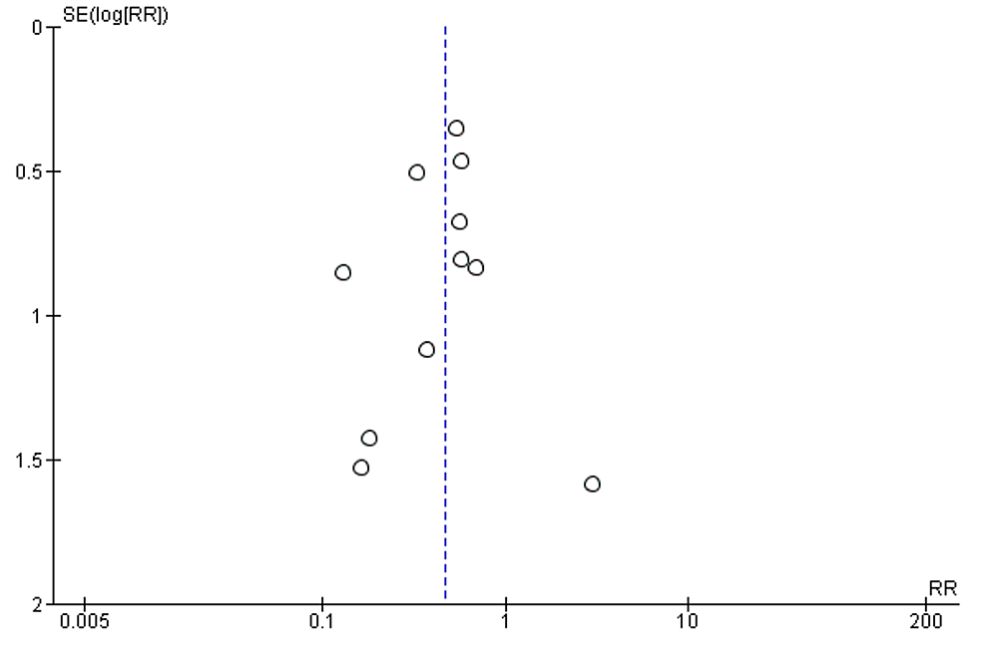
Funnel plot for 30-day mortality

The methodological quality assessment of included studies showed that all 11 studies were of good quality (Appendix C). The funnel plot for the publication bias is given below (Figure 2).

Result of Meta-Analysis

**Figure 3:**
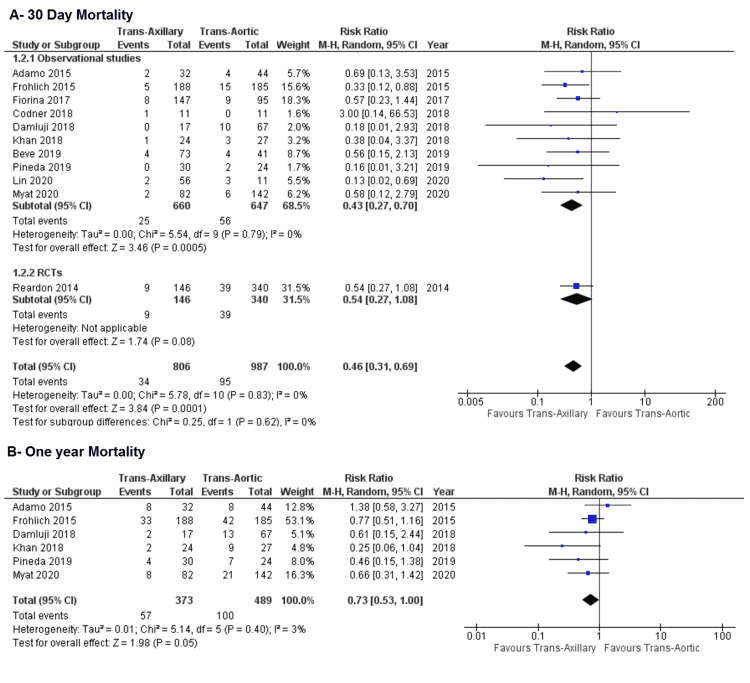
Forest plot for (A) 30-day mortality and (B) one-year mortality References: Reardon et al. (2014) [21], Adamo et al. (2015) [20], Fröhlich et al. (2015) [19], Fiorina et al. (2017) [18], Codner et al. (2018) [16], Damluji et al. (2018) [17], Khan et al. (2018) [5], Beve et al. (2019) [15], Pineda et al. (2019) [14], Lin et al. (2021) [13], and Myat et al. (2020) [12].

Mortality: All studies in our meta-analysis compared the 30-day mortality in both TAx and TAo approaches for TAVI. Pooling the estimates revealed that TAx group has significantly lower 30-day mortality compared to the TAo group (4.2% vs. 9.6%; RR: 0.46; 95% CI: 0.31, 0.69; P = 0.0001; Figure 3A) with no heterogeneity (I² = 0%). Out of 11 studies, only six studies reported data on one-year mortality. Although one-year mortality incidence was lower in the TAx group compared to the TAo group (15.3% vs. 20.4%), our cumulative findings revealed that this difference was not significant (RR: 0.73; 95% CI: 0.53, 1.00; P = 0.05; Figure 3B). These findings from observational studies and RCTs were consistent (P‐value for subgroup differences = 0.62). In addition, the heterogeneity level was low (I² = 3%).

**Figure 4:**
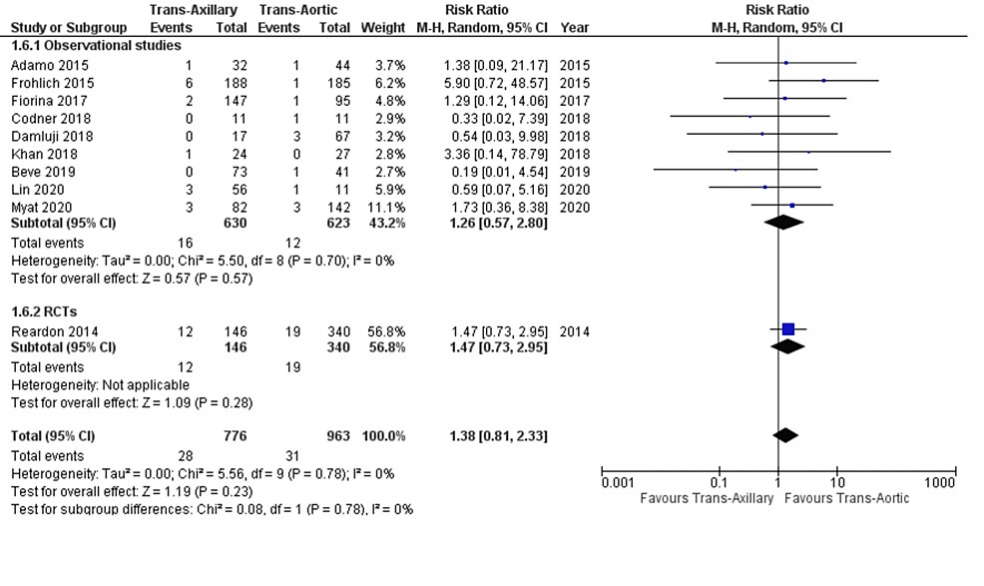
Forest plot for 30-day stroke References: Reardon et al. (2014) [21], Adamo et al. (2015) [20], Fröhlich et al. (2015) [19], Fiorina et al. (2017) [18], Codner et al. (2018) [16], Damluji et al. (2018) [17], Khan et al. (2018) [5], Beve et al. (2019) [15], Lin et al. (2021) [13], and Myat et al. (2020) [12].

Thirty-day stroke: Ten studies reported the incidence of 30-day stroke after TAVI. The incidences were reasonably similar (3.2% for TAo and 3.6% for TAx). The differences between the groups are not statistically significant (RR: 1.38; 95% CI: 0.81, 2.33; P = 0.23; Figure 4). The heterogeneity was low (I² = 0%). This finding was seen in both observational studies and RCTs (P‐value for subgroup differences = 0.78).

**Figure 5:**
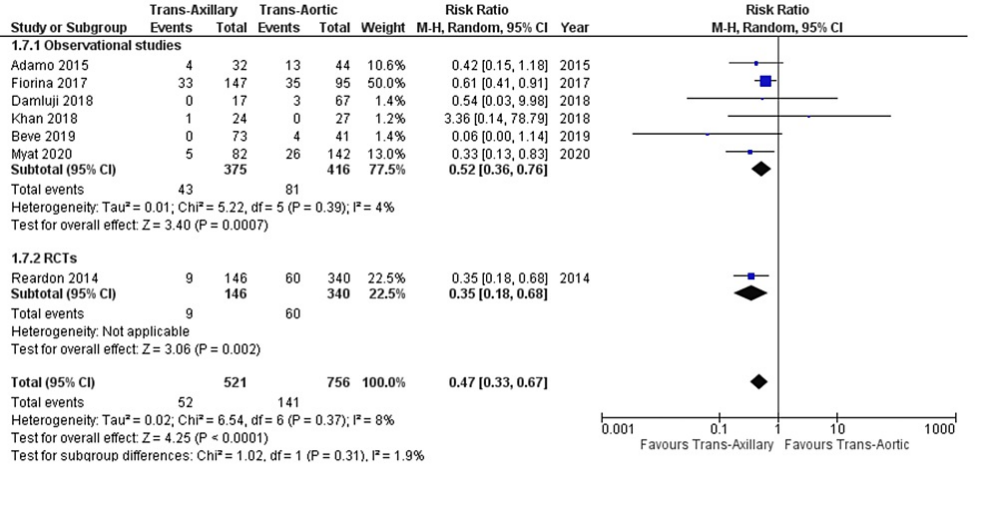
Forest plot for acute kidney injury References: Reardon et al. (2014) [21], Adamo et al. (2015) [20], Fiorina et al. (2017) [18], Damluji et al. (2018) [17], Khan et al. (2018) [5], Beve et al. (2019) [15], and Myat et al. (2020) [12].

AKI: Seven studies included in our analysis reported the data on AKI after TAVI. Stratifying the data for AKI revealed that the TAx approach was associated with a lower incidence compared to the TAo approach (9.9% vs. 18.6%; RR: 0.47; 95% CI: 0.33, 0.67; P < 0.0001; Figure 5) with a low heterogeneity (I² = 8%). These findings were consistent in observational studies and RCTs (P-value for subgroup = 0.31).

**Figure 6:**
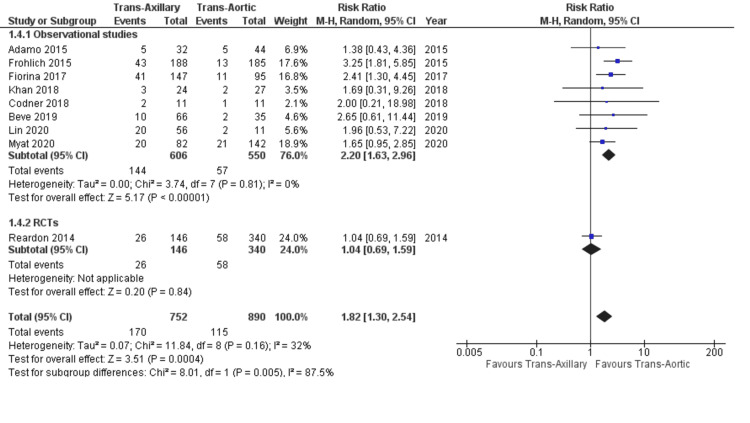
Forest plot for permanent pacemaker implantation References: Reardon et al. (2014) [21], Adamo et al. (2015) [20], Fröhlich et al. (2015) [19], Fiorina et al. (2017) [18], Khan et al. (2018) [5], Codner et al. (2018) [16], Beve et al. (2019) [15], Lin et al. (2021) [13], and Myat et al. (2020) [12].

Pacemaker implantation: Nine studies reported the incidence of pacemaker implantation after TAVR. The TAx approach was associated with a significantly higher incidence of implanting a supportive PPM than the TAo group (22.6% vs. 12.9%; RR: 1.82; 95% CI: 1.30, 2.54; P = 0.0004; Figure 6). The heterogeneity was low (I² = 32%). This finding was only consistent with the subgroup of observational studies (P-value for subgroup difference = 0.05).

**Figure 7:**
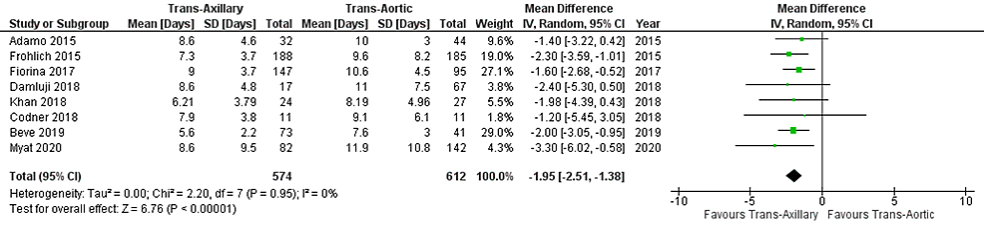
Forest plot for the length of hospital stay References: Adamo et al. (2015) [20], Fröhlich et al. (2015) [19], Fiorina et al. (2017) [18], Damluji et al. (2018) [17], Khan et al. (2018) [5], Codner et al. (2018) [16], Beve et al. (2019) [15], and Myat et al. (2020) [12].

Length of hospital stay: Eight studies included in our analysis reported the data on the length of hospital stay. The aggregated results showed significantly shorter LOS in TAx approach compared to the TAo approach (mean difference: −1.95; 95% CI: −2.51, −1.38; P < 0.00001; Figure 7) with no heterogeneity amongst studies (I² = 0%).

**Figure 8:**
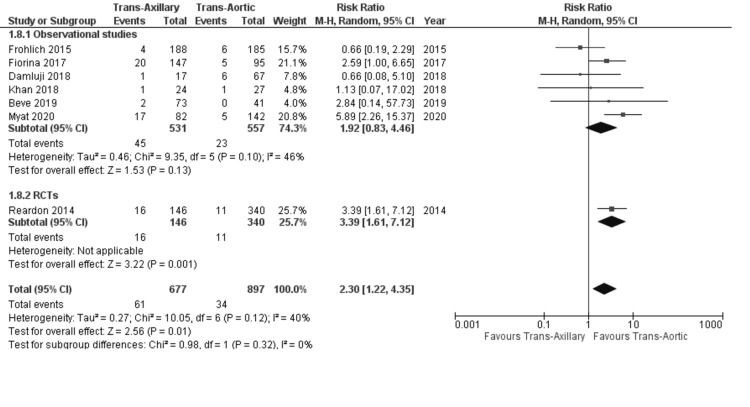
Forest plot for vascular complications References: Reardon et al. (2014) [21], Fröhlich et al. (2015) [19], Fiorina et al. (2017) [18], Damluji et al. (2018) [17], Khan et al. (2018) [5], Beve et al. (2019) [15], and Myat et al. (2020) [12].

Vascular complications: Seven out of 11 selected articles reported the incidence of vascular complications after TAVI. The pooled results were in favor of the TAo approach as the rate of vascular complications was higher in the TAx approach (9.0% vs. 3.8%; RR: 2.30; 95% CI: 1.22, 4.35; P = 0.01; Figure 8) with low heterogeneity between studies (I² = 40%). These findings were consistent with the RCTs subgroup. However, there was no statistically significant difference between the two subgroups (P-value for subgroup difference = 0.32).

**Figure 9:**
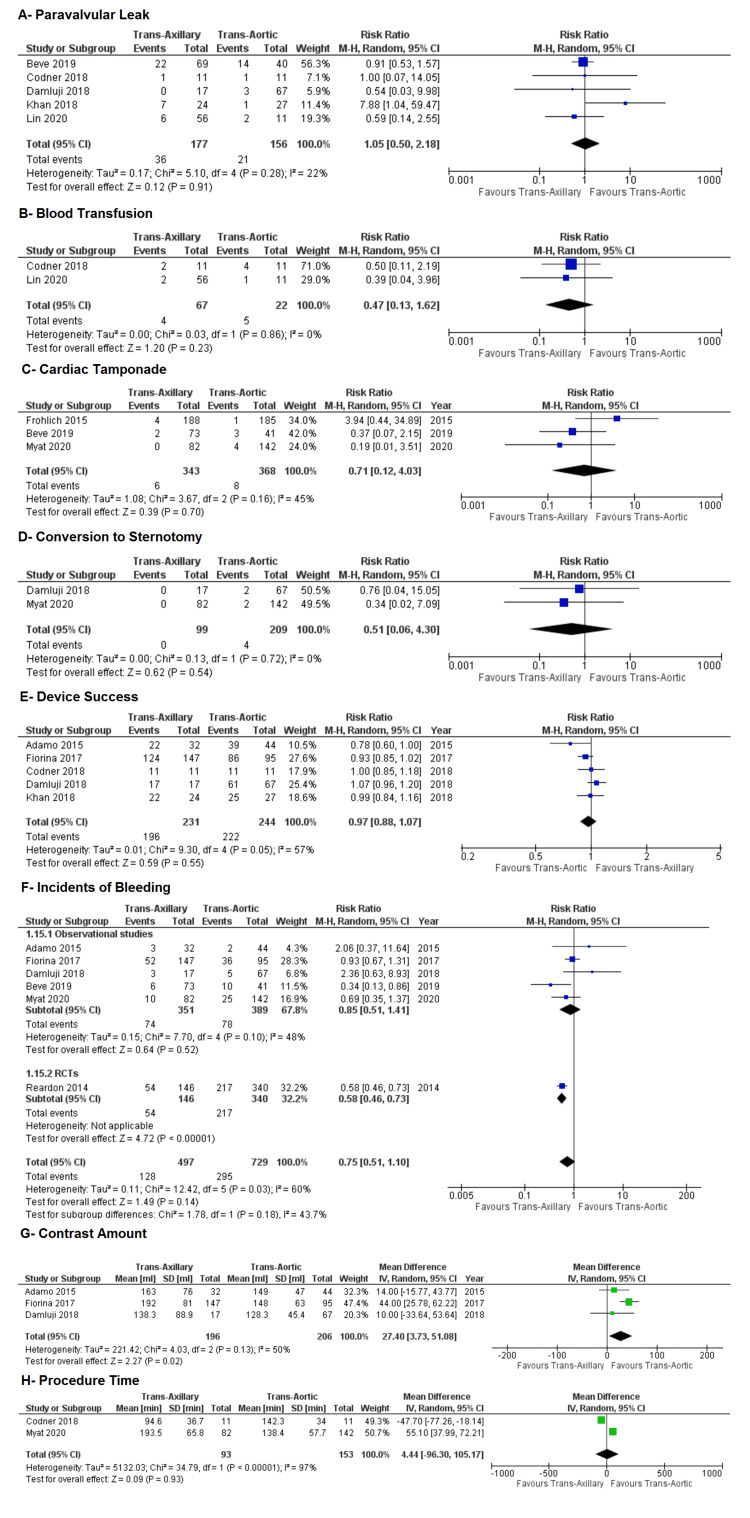
Forest plots for the following outcomes: (A) paravalvular leak, (B) blood transfusion, (C) cardiac tamponade, (D) conversion to sternotomy, (E) device success, (F) incidents of bleeding, (G) contrast amount, and (H) procedure time References: Reardon et al. (2014) [21], Adamo et al. (2015) [20], Fröhlich et al. (2015) [19], Fiorina et al. (2017) [18], Codner et al. (2018) [16], Damluji et al. (2018) [17], Khan et al. (2018) [5], Beve et al. (2019) [15], Lin et al. (2021) [13], and Myat et al. (2020) [12].

Other outcomes: The pooled results did not show any significant difference in the incidence of PVL (RR: 1.05; 95% CI: 0.50, 2.18; P = 0.91; Figure 9A) (I² = 22%), blood transfusion (RR: 0.47; 95% CI: 0.13, 1.62; P = 0.23; Figure 9B) (I² = 0%), tamponade (RR: 0.71; 95% CI: 0.12, 4.03; P = 0.70, Figure 9C) (I² = 45%), and conversion to sternotomy (RR: 0.51; 95% CI: 0.06, 4.30; P = 0.54; Figure 9D) (I² = 0%) between both groups (Figure 9).

**Figure 10:**
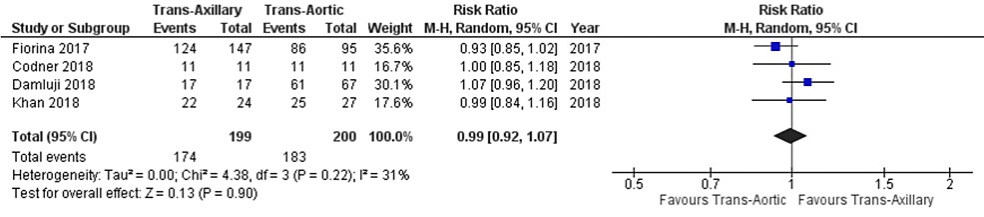
Forest plot for device success (sensitivity analysis) References: Fiorina et al. (2017) [18], Codner et al. (2018) [16], Damluji et al. (2018) [17], and Khan et al. (2018) [5].

Regarding device success, there was no significant difference observed between both approaches (RR: 0.97; 95% CI: 0.88, 1.07; P = 0.55; Figure 9E), with heterogeneity being of moderate nature (I² = 57%). We performed a sensitivity analysis to lower the heterogeneity to an acceptable level (I² = 31%), which still depicted no significant difference in device success between the approaches (RR: 0.99; 95% CI: 0.92, 1.07; P = 0.90; Figure 10).

**Figure 11:**
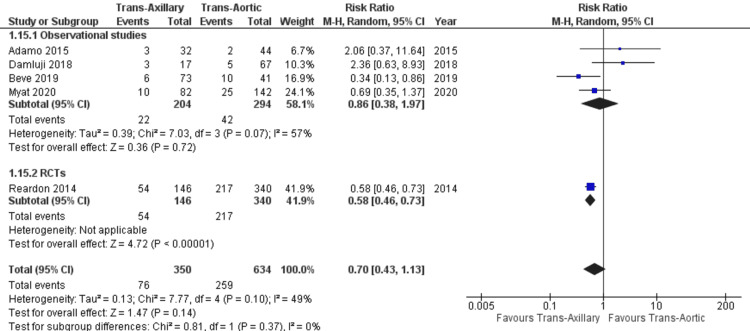
Forest plot for incidents of bleeding (sensitivity analysis) References: Reardon et al. (2014) [21], Adamo et al. (2015) [20], Damluji et al. (2018) [17], Beve et al. (2019) [15], and Myat et al. (2020) [12].

Similarly, no significant difference was found in the incidence of bleeding between both groups (RR: 0.75; 95% CI: 0.51, 1.10; P = 0.14; Figure 9F), with heterogeneity being of moderate nature (I² = 60%). Heterogeneity was decreased using sensitivity analysis (I² = 49%), and the pooled estimates remained insignificant (RR: 0.70; 95% CI: 0.43, 1.13; P = 0.14; Figure 11).

Three studies in our analysis reported data concerning the contrast amount used in the TAVI procedure. The TAo route used significantly less contrast amount as compared to the TAx approach (mean difference: 27.40; 95% CI: 3.73, 51.08; P = 0.02; Figure 9G) with moderate heterogeneity in the studies (I² = 50%).

Additionally, two studies reported procedure time. The cumulative results revealed no significant mean difference between the groups (mean difference: 4.44; 95% CI: −96.30, 105.17; P = 0.93; Figure 9H), with high heterogeneity amongst studies (I² = 97%).

Discussion

Femoral access, the conventional approach to TAVI, is associated with significantly fewer adverse outcomes than other approaches [4]. Even so, in less than half of the cases, it is denied due to complicated anatomies, such as small body habitus and severe peripheral vascular disease. In these cases, non-femoral techniques such as transapical (TA), TAo, and TAx approaches are considered. This updated meta-analysis compares two such techniques, i.e., the TAx versus TAo approach.

Our meta-analysis showed that the TAx had statistically significant lower 30-day mortality than the TAo approach, a finding also concluded by a previous meta-analysis [22]. This is in contrast to Myat et al., Pineda et al., and Beve et al., where there was no significant difference in in-hospital, 30-day, and one-year all-cause mortality [12,14,15]. It is speculated that this is related to the invasiveness between the two approaches, with TAx being less invasive than TAo and thus favoring a better outcome. Differences in the patients' baseline characteristic profile may also have been contributory, with TAo having individuals with higher comorbid factors including hypertension, diabetes mellitus, prior myocardial infarction, chronic obstructive pulmonary disease (COPD), peripheral vascular disease, higher Society of Thoracic Surgeons (STS) score, and higher logistic European System for Cardiac Operative Risk Evaluation (EuroSCORE) (shown in Table 1). The higher mortality score predicted by STS and EuroSCORE for the TAo group was, however, not significant. With respect to patient comorbidities, similar reasoning has been cited in the previous literature, notably one of the most extensive studies assessing the outcome so far [19].

Furthermore, the TAVI approach has been linked with an overall higher 30-day stroke incidence than traditional surgical replacement. This has been made evident by the appearance of new, clinically silent cerebral lesions on post-procedure MRI [23]. The likely reason for this is the dislodged atherosclerotic or calcific debris from the aorta or the calcified aortic valve [23]. Our meta-analysis evaluated the 30-day stroke incidence between the TAx and TAo approaches. The results concluded that no technique was superior when considering this particular complication. Considering the pooled estimates of studies included in this meta-analysis, the frequency was reasonably similar between the two approaches (28/776 events in TAx and 31/963 events in TAo). Prior atrial fibrillation has been cited as a risk factor for the development of stroke post-TAVR [24]. Since both approaches recorded a fairly similar incidence of prior atrial fibrillation (2.4% for TAx vs. 2.0% for TAo), this may also have been a contributing factor to the similar stroke rates between the two approaches [24]. However, another plausible explanation for these results may be the inadequate sample size of the included studies.

One other complication of TAVI is AKI. It is possibly caused by prerenal azotemia and nephrotoxic injuries, leading to renal ischemia and acute tubular necrosis (ATN) [25]. Renal ischemia is also attributed to hypovolemia, hemorrhage, low cardiac output, or renal vasoconstriction caused by vasoconstrictive medication [25]. When focusing on the non-femoral approaches and the AKI events associated with each approach, our results highlighted that the TAx approach was significantly safer than the TAo approach (9.9% vs. 18.6%). Despite lower contrast media being used in the TAo group, the incidence of AKI was much higher in the TAo group. This finding was also corroborated by Fiorina et al., where baseline creatinine levels were similar amongst the patient populations of the two approaches [18]. In a study conducted by Aregger et al., it was noted that amongst patients undergoing TAVI, higher rates of AKI were linked to lower hemoglobin concentration, higher blood transfusions rates, higher post-procedure thrombocytopenia, higher leukocyte count due to ongoing severe inflammatory response syndrome (SIRS), and an increased length of hospital stay [26]. Moreover, hypertension, diabetes, peripheral arterial disease, higher STS, and higher EuroSCORE have all been identified as factors associated with increased risk of AKI in patients undergoing TAVR [27]. With respect to our study, while many factors could not be ascertained due to paucity of the available data, the incidence of diabetes mellitus, hypertension, peripheral arterial disease, and mortality predictive scores was lower in the TAx group and these may have been crucial contributing reasons why lower AKI rates were observed in that group.

PPM implantation remains one of the frequent complications of TAVI. Conduction abnormalities originating from anatomic interaction between the valve prosthesis and the atrioventricular node and bundle of His are the implicated causes requiring pacemakers’ implantation [28]. According to a meta-analysis conducted by Zhan et al., male sex, baseline atrioventricular conduction delays, intra-procedural atrioventricular block, and use of mechanically expandable or self‐expanding prosthesis serve as positive predictors of PPM implantation in patients undergoing TAVI [29]. Furthermore, with respect to comorbidities, literature has reported diabetes as a significant predictor while hypertension, COPD, prior percutaneous coronary intervention (PCI), or prior aortic valve procedures were not [30]. Our meta-analysis concluded that the TAx approach had a higher pacemaker implantation rate. One possible confounder for this could be that most patients undergoing the TAx have self-expandable valves implanted, which are linked to higher rates of PPM placement [22]. This higher rate is attributed to the difference in design and frame of the valve that exerts a radial force on the conduction tissue [30]. However, the proximity of aortic access to the aortic valve annulus in the TAo approach possibly mediates a lesser risk of conduction interruption and ensures more precise valve placement.

When stratified for the length of hospital stay, our analysis concluded that the TAx group was associated with a shorter stay than the TAo group. This finding coincides with the previous meta-analysis [22]. The favorable outcomes in the TAx approach could be due to a less invasive surgical cut-down than the TAo approach, leaving the chest cavity untouched. Similarly, the requirement for ventilation and intensive care unit stay and duration of general anesthesia might also be less in TAx than TAo approach [19]. Literature on healthcare optimization has shown that patients may be optimized with reduced procedural times, and the risk of in-hospital infections is reduced with a shorter hospital stay [16]. Furthermore, same-day discharge and next-day discharge in patients undergoing uncomplicated TAVI have been associated with lower mortality, stroke, and 30-day rehospitalization [31].

Moreover, vascular complications are one of the significant concerns of TAVI due to the predominant use of large-bore sheaths for vascular access [32]. Major complications include aortic dissection, aortic rupture, annulus rupture, access site vascular injury, distal embolization, and ipsilateral lower extremity ischemia [32]. The rate of vascular complications was higher in the TAx approach in our analysis, which is consistent with the literature [33]. One reason could be that in a TAx TAVI, the proximal third of the axillary artery can be feasibly punctured in a fully percutaneous approach [32]. In this case, the minimum vessel diameter should be 6 mm, but it can exceed 7 mm in cases of prior coronary artery bypass grafting (CABG) surgery using the ipsilateral internal mammary artery [32]. The pattern of vascular complications in TAx TAVI is similar to that seen in the transfemoral approach. However, it is relatively difficult to achieve hemostasis with manual compression. This is due to the lack of supporting structures to reinforce during compression at the TAx site [32]. On the contrary, the TAo approach can lead to tearing suture lines and an incommodious arterial closure due to the fragility of ascending aorta.

Among the other outcomes evaluated in this meta-analysis, the pooled estimates showed no statistically significant difference between TAx and TAo approaches to TAVI in the incidence of PVL, blood transfusion, tamponade, conversion to sternotomy, bleeding, device success, contrast amount, and procedure time. Inadequate sample size and heterogeneity might have affected these results. In addition, the sensitivity analysis did not reveal a significant difference amongst the outcomes showing moderate/high heterogeneity. Although most of the outcomes were in favor of TAx TAVI, it is too early to say if it would be better than TAo TAVI. More studies, especially RCTs, are recommended to monitor the sequelae of both approaches.

The major limitation of our study stems from the small number of studies that qualified for the meta-analysis and only one available RCT. These studies involve self-expandable and balloon-expandable valves of different generations, and with the mixed-use of devices, the small number of studies did not allow a device stratification. Therefore, our study could be confounded by a device-related bias. In addition, most of the studies included were unmatched retrospective cohort studies. Although these studies reported outcomes using the Valve Academic Research Consortium (VARC) criteria, data reporting still has significant heterogeneity, including baseline characteristics and outcome measures. There could be inherent publication bias with the present meta-analysis due to the nature of retrospective studies that tend to report favorable outcomes. These necessitate improved data collection and standardization of the health centers to remove the surgical bias [19], and studies should also focus on other aspects of these procedures (e.g. procedural success and rate of re-operations). To authenticate the findings concluded in this meta-analysis and further improve our understanding of the efficacy, safety, and risk profile between TAx and TAo approaches for TAVI, large sample randomized clinical trials are required on a wide scale.

## Conclusions

To our knowledge, this meta-analysis is the largest to date consisting of 1793 patients, directly comparing the TAx and TAo techniques for AVR. We observed that the TAx approach had a more favorable profile regarding outcomes such as lower 30-day mortality, lower incidence of AKI, and shorter hospital stay. In others, TAo reported a better result, for instance, lower incidence of PPM implantation and lesser vascular complications. Other outcomes such as conversion to sternotomy, paravalvular leak, blood transfusion, procedure time, and contrast amount had no significant differences, regardless of the technique used. Truly reaching a definite verdict regarding the better technique overall will require further studies with rigorous data collection and standardization. However, at present, this meta-analysis provides physicians with an in-depth evaluation of the advantages and disadvantages of each method, guiding their decisions according to the outcomes desired in each patient.

## Appendices

Appendix A

**Table 2:** Search strategy

Search strategy
("transcatheter aortic valve replacement"[MeSH Terms] OR ("transcatheter"[All Fields] AND "aortic"[All Fields] AND "valve"[All Fields] AND "replacement"[All Fields]) OR "transcatheter aortic valve replacement"[All Fields] OR (("percutaneous"[All Fields] OR "percutaneously"[All Fields] OR "percutaneous"[All Fields]) AND ("aorta"[MeSH Terms] OR "aorta"[All Fields] OR "aortic"[All Fields] OR "aortics"[All Fields])) OR ("transcatheter aortic valve replacement"[MeSH Terms] OR ("transcatheter"[All Fields] AND "aortic"[All Fields] AND "valve"[All Fields] AND "replacement"[All Fields]) OR "transcatheter aortic valve replacement"[All Fields] OR ("transcatheter"[All Fields] AND "aortic"[All Fields] AND "valve"[All Fields] AND "implantation"[All Fields]) OR "transcatheter aortic valve implantation"[All Fields]) OR "TAVI"[All Fields] OR "TAVR"[All Fields]) AND ("axilla"[MeSH Terms] OR "axilla"[All Fields] OR "axillary"[All Fields] OR "axillaries"[All Fields] OR "axillaris"[All Fields] OR "Transaxillary"[All Fields] OR "Trans-axillary"[All Fields] OR ("subclavian"[All Fields] OR "subclavians"[All Fields]) OR "Trans-subclavian"[All Fields] OR ("transcervical"[All Fields] OR "transcervically"[All Fields])) AND ("transthoracal"[All Fields] OR "transthoracic"[All Fields] OR "transthoracical"[All Fields] OR "transthoracically"[All Fields] OR (("direct"[All Fields] OR "directed"[All Fields] OR "directing"[All Fields] OR "direction"[All Fields] OR "directional"[All Fields] OR "directions"[All Fields] OR "directivities"[All Fields] OR "directivity"[All Fields] OR "directs"[All Fields]) AND ("aorta"[MeSH Terms] OR "aorta"[All Fields] OR "aortic"[All Fields] OR "aortics"[All Fields])) OR "trans-aortic"[All Fields] OR ("transaortal"[All Fields] OR "transaortic"[All Fields]) OR (("direct"[All Fields] OR "directed"[All Fields] OR "directing"[All Fields] OR "direction"[All Fields] OR "directional"[All Fields] OR "directions"[All Fields] OR "directivities"[All Fields] OR "directivity"[All Fields] OR "directs"[All Fields]) AND ("aorta"[MeSH Terms] OR "aorta"[All Fields] OR "aortic"[All Fields] OR "aortics"[All Fields])))

Appendix B

**Table 3:** Newcastle-Ottawa Scale for quality assessment

Newcastle-Ottawa Scale										
Author	Year	Selection				Comparability	Outcome			Total score
		Representativeness of the exposed cohort	Selection of the non-exposed cohort	Ascertainment of exposure	Demonstration that the outcome of interest was not present at the start of the study	Comparability of cohorts on the basis of the design or analysis	Assessment of outcome	Was follow-up long enough for outcomes to occur	Adequacy of follow up of cohorts	
Damluji et al. [17]	2018	⋆	-	⋆	⋆	⋆⋆	⋆	⋆	⋆	8
Fiorina et al. [18]	2017	⋆	-	⋆	⋆	⋆	⋆	⋆	⋆	7
Fröhlich et al. [19]	2015	⋆	-	⋆	⋆	⋆⋆	⋆	⋆	⋆	8
Khan et al. [5]	2018	⋆	-	⋆	⋆	⋆⋆	⋆	⋆	⋆	8
Adamo et al. [20]	2015	⋆	-	⋆	⋆	⋆⋆	⋆	⋆	⋆	8
Beve et al. [15]	2019	⋆	-	⋆	⋆	⋆	⋆	⋆	⋆	7
Codner et al. [16]	2018	⋆	-	⋆	⋆	⋆⋆	⋆	-	⋆	7
Lin et al. [13]	2021	⋆	-	⋆	⋆	⋆	⋆	⋆	⋆	7
Myat et al. [12]	2020	⋆	-	⋆	⋆	⋆⋆	⋆	⋆	⋆	8
Pineda et al. [14]	2019	⋆	-	⋆	⋆	⋆	⋆	⋆	⋆	7
Reardon et al. [21]	2014	⋆	-	⋆	⋆	⋆	⋆	⋆	⋆	7

Appendix C

**Table 4:** Outcomes after sensitivity analysis for the possible overlapping studies

Outcomes	No. of studies	Risk ratio (RR)/weighted mean difference (WMD), 95% CI	Heterogeneity (%)	P-value
30-day mortality	10	0.45 (0.30, 0.68)	0	0.0001
30-day stroke	9	1.38 (0.81, 2.35)	0	0.24
Permanent pacemaker implantation	8	1.87 (1.30, 2.71)	40	0.0008
Acute kidney injury	6	0.46 (0.29, 0.72)	23	0.0007
Length of hospital stay	7	−2.01 (−2.60, −1.41)	0	<0.00001
Device success	4	0.99 (0.92, 1.07)	31	0.22
Contrast amount	2	33.02 (1.86, 64.18)	50	0.04
Incidents of bleeding	5	0.71 (0.48, 1.05)	63	0.08
